# Integrated analysis of RNA methylation regulators crosstalk and immune infiltration for predictive and personalized therapy of diabetic nephropathy

**DOI:** 10.1186/s40246-023-00457-9

**Published:** 2023-02-10

**Authors:** Jia Li, Dongwei Liu, Jingjing Ren, Guangpu Li, Zihao Zhao, Huanhuan Zhao, Qianqian Yan, Jiayu Duan, Zhangsuo Liu

**Affiliations:** 1 Research Institute of Nephrology, Zhengzhou University, Zhengzhou, 450052 People’s Republic of China; 2 Traditional Chinese Medicine Integrated Department of Nephrology, The First Affiliated Hospital of Zhengzhou University, Zhengzhou, 450052 People’s Republic of China; 3 Henan Province Research Center for Kidney Disease, Zhengzhou, 450052 People’s Republic of China; 4 Key Laboratory of Precision Diagnosis and Treatment for Chronic Kidney Disease in Henan Province, Zhengzhou, 450052 People’s Republic of China

**Keywords:** RNA methylation, Epigenetics, Diabetic nephropathy, Immune profiles, Immunotherapy

## Abstract

**Background:**

RNA methylation is a widely known post-transcriptional regulation which exists in many cancer and immune system diseases. However, the potential role and crosstalk of five types RNA methylation regulators in diabetic nephropathy (DN) and immune microenvironment remain unclear.

**Methods:**

The mRNA expression of 37 RNA modification regulators and RNA modification regulators related genes were identified in 112 samples from 5 Gene Expression Omnibus datasets. Nonnegative Matrix Factorization clustering method was performed to determine RNA modification patterns. The ssGSEA algorithms and the expression of human leukocyte antigen were employed to assess the immune microenvironment characteristics. Risk model based on differentially expression genes responsible for the modification regulators was constructed to evaluate its predictive capability in DN patients. Furthermore, the results were validated by using immunofluorescence co-localizations and protein experiments in vitro.

**Results:**

We found 24 RNA methylation regulators were significant differently expressed in glomeruli in DN group compared with control group. Four methylation-related genes and six RNA regulators were introduced into riskScore model using univariate Logistic regression and integrated LASSO regression, which could precisely distinguish the DN and healthy individuals. Group with high-risk score was associated with high immune infiltration. Three distinct RNA modification patterns were identified, which has significant differences in immune microenvironment, biological pathway and eGFR. Validation analyses showed the METTL3, ADAR1, DNMT1 were upregulated whereas YTHDC1 was downregulated in DN podocyte cell lines comparing with cells cultured by the normal glucose.

**Conclusion:**

Our study reveals that RNA methylation regulators and immune infiltration regulation play critical roles in the pathogenesis of DN. The bioinformatic analyses combine with verification in vitro could provide robust evidence for identification of predictive RNA methylation regulators in DN.

**Supplementary Information:**

The online version contains supplementary material available at 10.1186/s40246-023-00457-9.

## Background

Diabetic nephropathy (DN) is one of the most common microvascular complications of diabetes mellitus and the leading cause of end-stage renal disease (ESRD) [1]. The latest reports from Diabetes Atlas estimated the number of patients with diabetes mellitus (DM) is 463 million in 2019 and is predicted to reach 700 million by 2045 [2]. The exact mechanisms involved in the pathogenesis of DN is complicated and still not fully understood. Current therapies for DN mainly focus on controlling plasma glucose and blood pressure by using hypoglycemic agents and renin–angiotensin–aldosterone system (RAAS) blockers, which could only slow down the progression to ESRD but defeat to prevent it [3, 4]. Thus, novel therapeutic targets need to be discovered. A substantial body of evidence now indicated that the existence of epigenetic regulatory (e.g., DNA methylation and noncoding RNA) in the field of renal disease beyond the role of genetic in the pathogenesis of DN [5]. However, the identification of post-transcriptional epigenetic modification of RNA in DN is still unclear.

Like DNA methylation, RNA methylation modifications regulate gene expressions without alter the sequence of RNA and can influence the interaction between the genes and microenvironment. Increasing evidences demonstrate that genes associated with the progression of DN are regulated not only by traditional signaling pathways but also by epigenetic mechanisms. As a post-transcriptional modification, N6-methyladenosine (m6A), N1-methyladenosine (m1A), 5-methylcytosine (m5C), Alternative polyadenylation (APA), and adenosine-to-inosine (A-I) editing were the main RNA methylation modifications involving in RNA splicing [6], stability [7], translation [8], initiation of miRNA biogenesis [9] and subsequently affecting various physiological and pathological processes [10], such as cell differentiation, immune responses, epithelial-mesenchymal transformation.

RNA methylation has been proven to be related to human physiologies and its mis-regulation are linked with a variety of human diseases [11]. Previous study reported that the overexpression of METTL14, an m6A methyltransferase, contributed to extracellular matrix accumulation of renal tubular cells in diabetic kidney diseases via regulated PI3K/Akt signaling pathway [12]. Even though m6A modification in RNA were discovered in the 1950s, our understanding of RNA methylation modifications is limited in DN. Other RNA modifications within eukaryotic mRNA including m1A, m5C, APA, A-I editing, were barely researched in DN. m1A, as a reversible RNA modification, is methyl on the N1 position of adenosine and affect the translation of downstream genes and RNA–protein interaction [13]. m5C has emerged as the key regulators in modulating the translation and stability of RNA and ribosome assembly through its effector proteins- “writers” (methyltransferases), “readers,” and “erasers” (demethylase). Recent studies find that m5C are closely related to CD4^+^ T cells from systemic lupus erythematosus patients [14]. External mutation of a m5C methyltransferase NSUN3 lead to reduced mitochondrial translation, leading to severe multisystem mitochondrial diseases [15]. A-I editing is catalyzed by adenosine deaminases acting on RNA (ADARs) which binding to double-stranded RNA substrates [16]. Depletion of m6A-related enzymes increases the expression of ADAR enzymes resulting in upregulated A-I editing on the same m6A-depleted transcripts which showed a negative correlation between m6A and A-I [17]. APA generates distinct 3’ termini on mRNAs and other RNA polymerase II transcripts and affects mRNA length and stability.

Since these five RNA methylation patterns were crucial in the gene expression programing, immune cells regulatory and cell metastasis, identifying the epigenetic signatures in DN and complicated interrelations occurring between m6A and other well-known epigenetic modifications are urgently needed. In DN, RNA methylation not only affect the microenvironment but, importantly, mediate the persistent expression of DN-related genes and phenotypes induced by long-term hyper-glycemic exposure. However, previous studies on DN mainly focus on the specific amino acid residues of histone modification or DNA methylation [18]. The aberrant expression profiles of RNA methylation on DN need to be further analyzed. In our current study, we integrated the epigenetic features and immune characteristics in 111 samples from Gene Expression Omnibus (GEO) datasets. Then, nonnegative matrix factorization (NMF) clustering was used to identify three modification patterns and the correlation with immune landscape. A risk model was employed to further demonstrate the RNA methylation characteristics in DN. Finally, we combined cell lines with experimental techniques in vitro to validate the expression of these significant regulators. The bioinformatic analyses of epigenetic events during the early stages of DN could provide valuable evidences for discovering new therapeutic strategies and reducing disease burden on patients.

## Methods

### Data collection and processing

**Fig. 1:**
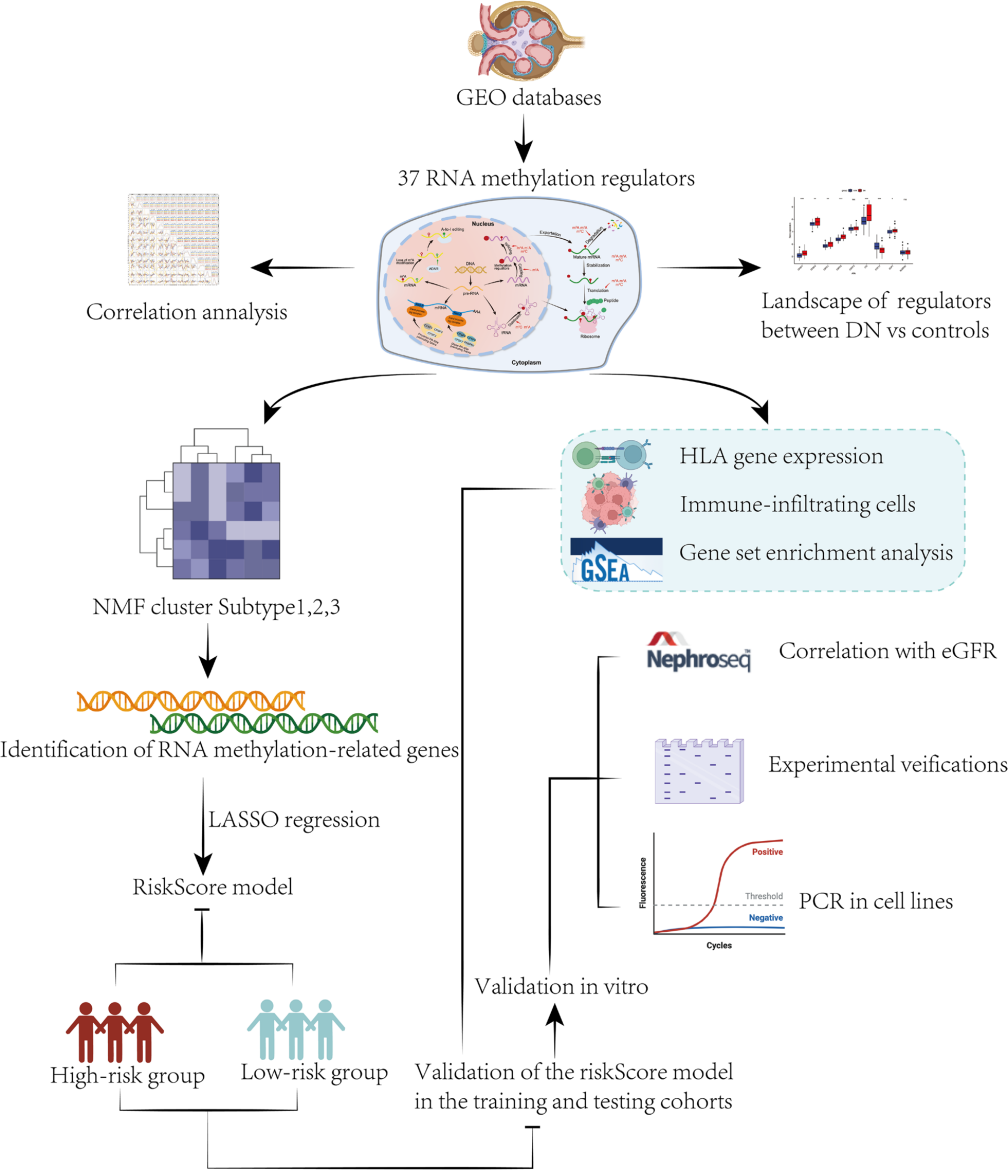
Schematic flowchart of this study

The flowchart of the present study is shown in Fig. 1. Gene expression data based on RNA sequencing were obtained from the Gene Expression Omnibus (GEO) datasets. Four eligible datasets (GSE99339, GSE41783, GSE30528, GSE104948) were combined. After excluding patients who had no gene expression data from glomeruli, 111 samples were enrolled for further analysis, including those from the GSE99339 (14 DN samples, 11 tumor nephrectomy controls), GSE41783 (14 DN samples, 17 tumor nephrectomy controls), GSE30528(9 DN samples, 13 controls), GSE104948(12 DN samples, 21 controls). Log scales matrix data was downloaded and then 37 RNA methylation regulators were collected according to the previously published literature [19–21]. The expression data of different datasets were normalized by using the "sva" R package (Version 3.46.0) for ComBat Batch correction to remove batch effects (Additional file 1: Fig. S1). The "limma" R package (Version 3.54.0) was applied to determine the differential expression of the RNA methylation regulators in the DN and tumor adjacent control samples. The interaction between RNA methylation regulators at the protein level was examined using Networkanalyst platform. The expression relationship among 37 regulators was evaluated by Pearson correlation analysis.

### Immune characteristics analysis

Single-sample gene set enrichment analysis (ssGSEA) algorithm was used and 28 gene sets labeling different immune-infiltrating cells were contained for further analysis [22]. Enrichment scores were calculated using ssGSEA in the R "GSVA" package (Version 1.42.0) characterizing the infiltration level of each type of immune cell in samples [23]. Immune reaction pathways were obtained from the ImmPort database. Correlation of RNA methylation regulators with immune-infiltrating cells, immune reaction pathways and HLA gene expression were calculated by spearman correlation analysis.

### Gene set variation analysis (GSVA) and Biological enrichment analysis

In order to investigate the variation in biological signaling pathways among different RNA methylation regulators, gene set variation analysis (GSVA) enrichment analysis with R ‘GSVA’ package were employed. Gene sets “h.all.v7.5.1.symbols.gmt,” “kegg.v7.5.1.symbols.gmt,” “biocarta.v7.5.1.symbols.gmt,” “reactome.v7.5.1.symbols.gmt,” and “wikipathways.v7.5.1.symbols.gmt” were downloaded from MSigDB database (v7.5.1). The gene set was considered significantly enriched if adjusted *P* value were less than 0.05. The R pheatmap package (Version 1.0.12) was used to visualize the results of HALLMARK and REACTOME pathways.

### Consensus functional clustering

Based on 37 RNA methylation regulators, Nonnegative Matrix Factorization (NMF) clustering was utilized to determine the optimal number and stability among all classifications. The *k* that before the highest variation in clustering was selected. The R package "NMF" (Version 0.24.0) with brunet algorithm and 100 nruns was performed for the consensus clustering. The RNA methylation regulators among three cluster subtypes and immune-infiltrating cells, immune reaction pathways and HLA gene expression were compared by ANOVA test.

### Construction and validation of risk model

The overlapping of differentially expressed genes (DEGs) among three distinct subtypes were identified as RNA methylation-related genes by using empirical Bayesian approach. The R package "limma" were used to evaluated DEGs between three different modification clusters. Twenty-four RNA methylation regulators were also introduced for the model construction. In total, 52 RNA regulatory genes for univariate logistic regression and the significant differently expressed genes were extracted. The R package "caret" (Version 6.0-93) was used to split data into training and test cohort. Identified genes related to prognosis using Least Absolute Shrinkage and Selection Operator (LASSO) regression to construct a risk model in the training cohort. The riskScore of each sample was separately quantified in the training and validation cohorts as follows:

$$\mathop \sum \limits_{i = 1}^{n} {\text{Coef}}_{i} *x_{i}$$

where *i* means the RNA regulatory genes and the coefficients were calculated form the LASSO regression algorithm. Then, High- and Low-risk groups were divided according to the median risk score. To characterize the immune-infiltrating cells and immune reaction pathways affected by risk score, we performed differential expression and correlation analysis to identify the immune characteristics which were significantly correlated with risk score.

### Correlation between eGFR correlation and RNA methylation regulatory genes

To evaluate the correlation between estimated Glomerular Filtration Rate (eGFR) and RNA methylation regulatory genes, we performed a web-based analysis with Nephroseq V5 tool and visualized data by using "ggplot2" R package (Version 3.4.0).

### Cell culture

Conditionally immortalized human podocyte cell line (HPC) and mouse podocyte cell line (MPC) were obtained from Jinling Clinical Medical College of Nanjing Medical University. The cells were cultured in RPMI-1640 (gibco, USA), 10% Fetal Bovine Serum (gibco), and penicillin/streptomycin medium at 33 °C in growth permissive conditions. IFN-γ was specially supplemented for MPC and Insulin-Transferrin-Selenium for HPC. HPC and MPC were treated with high glucose medium (HG) in a concentration of 30 mM glucose for 48 h when cells were cultured at 37 °C 7 days for differentiate. The normal concentration of glucose (5.5 mM) serves as control.

### RNA extraction and quantitative real-time PCR

Total cellular RNA was extracted using RNeasy Mini Kit (Qiagen, Hilden, Germany) in accordance with the manufacturer’s protocol. Complementary DNA (cDNA) was synthesized with RevertAid First Strand cDNA Synthesis Kit (Thermo Fisher Scientific, Shanghai, China). Quantitative Real-time PCR (qRT-PCR) was performed using the Fast Start Essential DNA Green Master (Roche, Shanghai, China). The primer for target mRNA was designed and synthesized by Sangon Biotech (Shanghai, China) (Additional file 2: Table S1). All experiments were performed in triplicate and the amplification signals of β-actin mRNA served as the internal control.

### Western blot analysis

Cultured cells were lysed and protein was extracted using radioimmunoprecipitation assay (RIPA) lysis buffer supplemented with protease inhibitor (CW2200S, CWBio, China) and phosphatase inhibitors (CW2383S, CWBio, China). Equal amounts of proteins were resolved by 10% or 6% SDS-PAGE and were subjected to immunoblot analysis using standard methods with the following antibodies: NHPS2 (ab50339, Abcam), Synaptopodin (21064-1-AP), METTL3 (96391 s, Cell Signaling), YTHDC1 (77422 s, Cell Signaling), ADAR1(bs-2168R, Bioss), DNMT1 (ab188453, Abcam), WTAP (60,188–1-Ig, Proteintech) (all used at a 1:1000) and β-actin(AP0060, Bioworld) (used at 1:5000). The Secondary antibodies were purchased from Dingguo Biotechnology (Beijing, China). Semiquantitative analysis of the protein density by western blotting was performed using ImageJ (Version 1.5.3).

### Immunofluorescence staining

HPC and MPC grown on coverslips were fixed with 4% paraformaldehyde for 10 min at room temperature, followed by permeabilization with 0.5% Triton X-100 for 10 min and blocked with 3% bovine serum albumin and immunostained with anti-METTL3 (ab195352, Abcam, 1:1000), ADAR1(bs-2168R, Bioss, 1:100), WTAP (60188-1-Ig, Proteintech, 1:100) or anti-DNMT1 (ab188453, Abcam, 1 μg/ml) overnight at 4 °C. After washing with PBS, HPC or MPC were incubated with Alexa Fluor 488 goat anti-rabbit IgG (Invitrogen, A32731, 1:200) and DAPI (Vectorlabs, H-1200) for nuclear staining. The specimens were visualized and analyzed by using Zeiss LSM880 confocal microscope (Carl Zeiss, Germany).

### Statistical analysis

All statistical analyses were performed using R version 4.1.2 and SPSS 24.0. The expression levels of the RNA methylation regulators were compared in DN samples versus controls using Wilcoxon rank-sum test. The normal distribution data was statistically analyzed by Student's-t test between two groups and more than two groups were performed by one-way ANOVA. Univariate logistic regression analyses were performed to determine the independent prognostic factors. *P* < 0.05 was considered has a statistically significant.

## Results

### Landscape of RNA methylation regulators in diabetic nephropathy

**Fig. 2:**
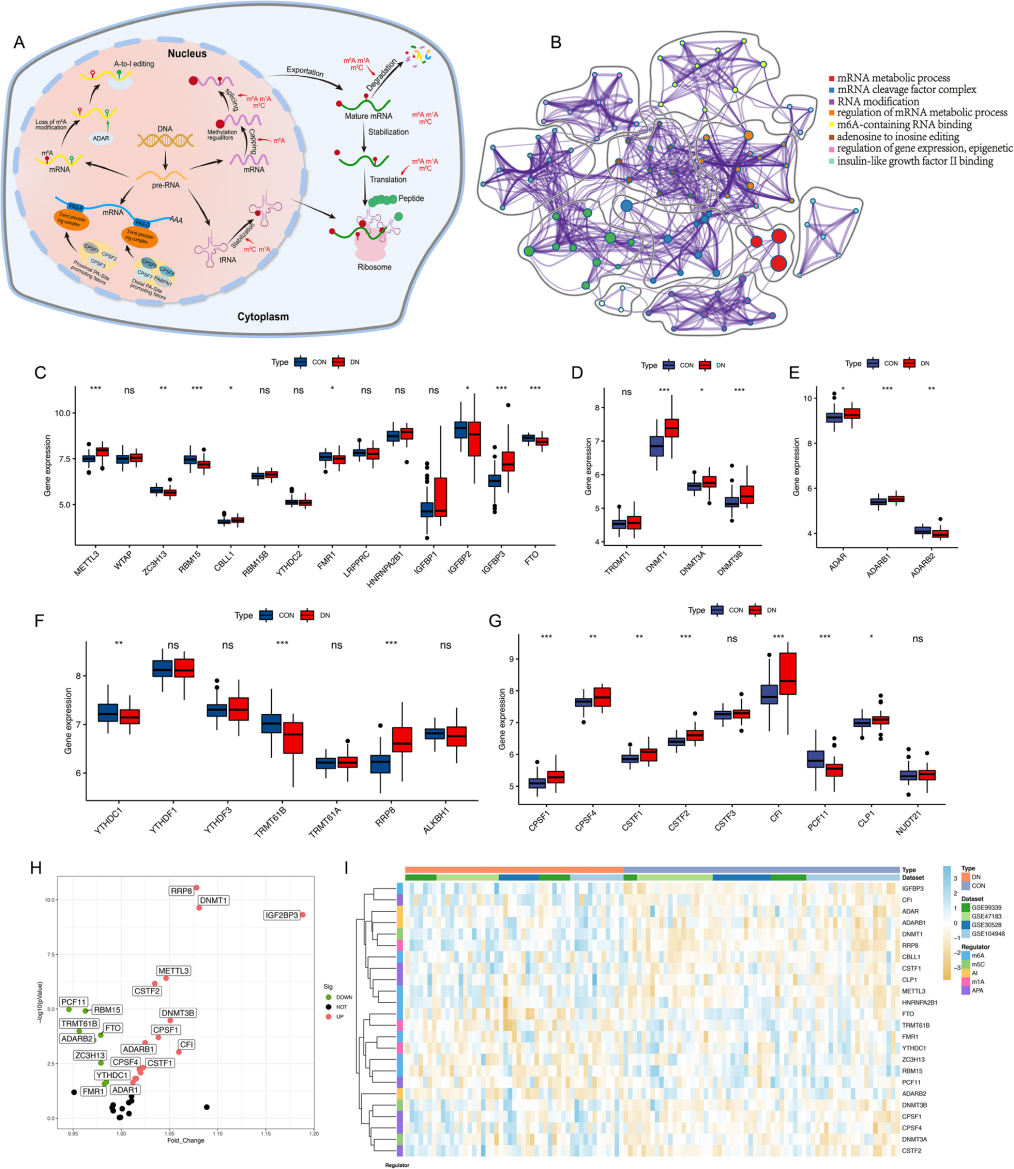
The landscape of RNA methylation regulators in diabetic nephropathy. **A** The overview of the metabolic process of RNA methylation regulation in cytoplasm. **B** Metascape enrichment network visualization showed the clusters and similarities of enriched terms. **C** The box plot demonstrated the transcriptome expression status of 14 m6A regulators between control and diabetic nephropathy samples. **D** Expression status of 4 m5C regulators. **E** Expression status of 3 A to I editing regulators. **F** Expression status of 7 m1A regulators. **G** Expression status of 9 APA regulators. **H** The volcano plot shows the summary of expression information of 37 RNA methylation regulators. **I** Difference in features and expression levels of RNA methylation regulators between control and diabetic nephropathy samples

A total of 37 RNA modification regulators (Additional file 2: Table S2) from five types of RNA modifications, including 14 m6A methylation regulators, 3 A-I methylation regulators, 4 m5C methylation regulators, 7 m1A methylation regulators, and 9 APA methylation regulators were identified. Figure 2A illustrates the biological processes and crosstalk between epigenetic modulators. The coordinated relationships between each epigenetic counterpart elicit the epigenetic remodeling, which accounts for the perplexing modulations of various bioprocesses. Metascape and GO enrichment were used to demonstrate how these associations impact biological functions, particularly enrichment in mRNA metabolic process (Fig. 2B). To analyze the distinct expression of the RNA modification regulators, we compared the mRNA expression of regulators between DN and control samples and found that a majority of m5C, A-I, and APA were significant highly expressed, whereas ZC3H13, RBM15, FMR1, IGF2BP2, FTO of m6A, ADARB2 of A-I, YTHDC1, TRMT61 of m1A, and PCF11 of APA were downregulated in DN samples (Fig. 2C–G).

**Fig. 3:**
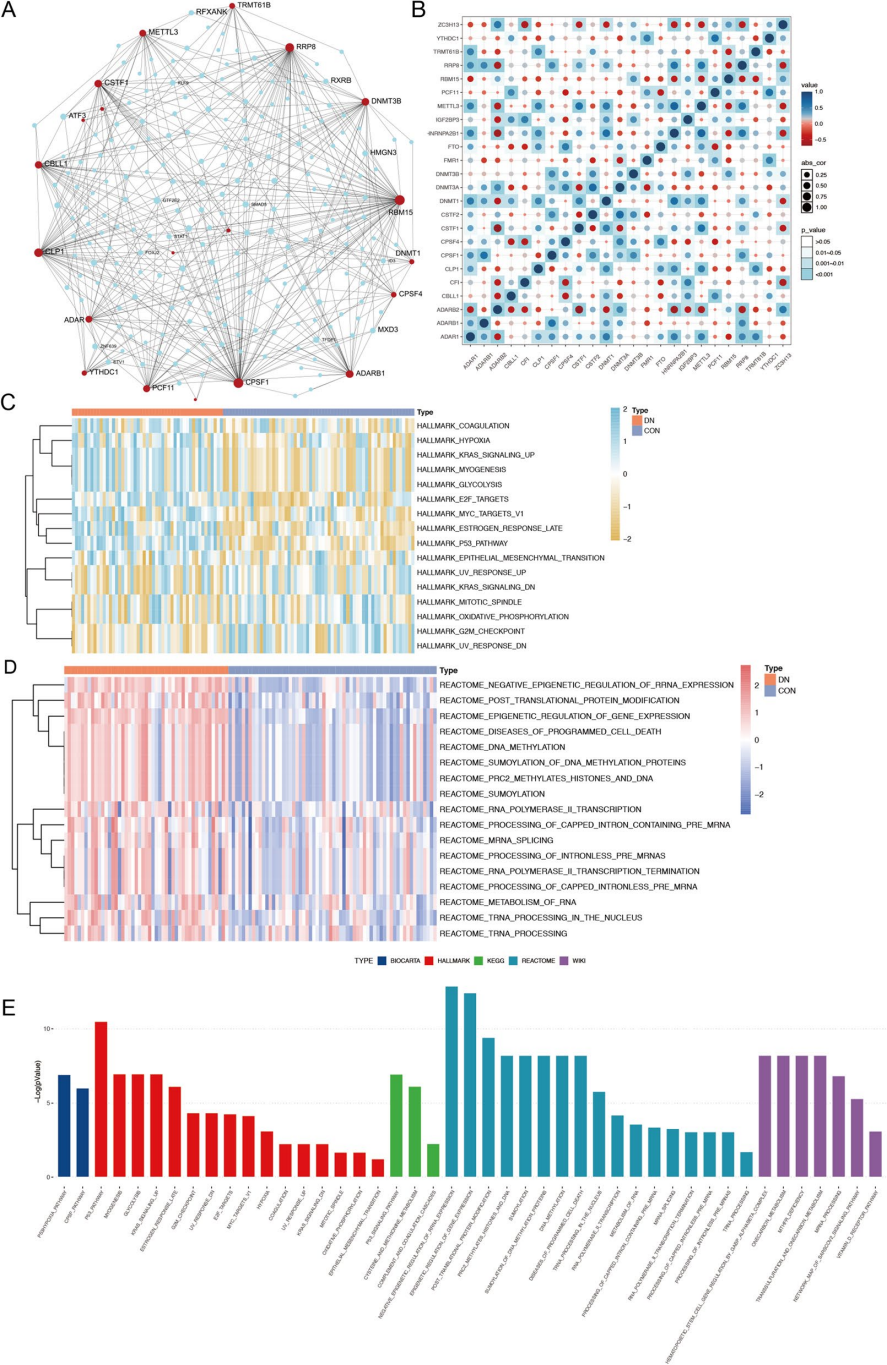
RNA methylation modification patterns and relevant biological pathways. **A** The interaction of expression on RNA methylation regulators in diabetic nephropathy. **B** Correlations among the expression of RNA methylation regulators in the meta-cohort. **C**, **D** Heatmap shows the GSVA score of representative Hallmark pathways and Reactome pathways between control and diabetic nephropathy samples. **E** Box plot shows the BIOCARTA, HALLMARK, KEGG, REACTOME, and WIKI enrichment analyses of RNA methylation regulators between control and diabetic nephropathy samples

The intersection of expression on 24 RNA methylation regulators is shown in Fig. 3A. The Pearson correlation analysis showed that m6A “reader” METTL3 and HNRNPA2B1 were significant positively correlated with a value of 0.73, whereas A-I regulator ADAR2 and APA regulator CSTF1 were most negatively correlated with a value of − 0.69 (Fig. 3B, Additional file 2: Table S3). The results demonstrated that the RNA modification regulators were not only correlated in same modification types, but also had relationships with other different modification types. Next, GSVA was adopted to evaluate the biological pathways of these distinct RNA modification types. They were differently enriched in HALLMARKS pathway including glycolysis, KRAS signaling upregulation, P53 pathway, epithelial-mesenchymal transition, and oxidative phosphorylation in DN samples (Fig. 3C). In addition, REACTOME pathways, including negative epigenetic regulation of rRNA expression, post translation protein modification and diseases of programmed cell death, were precisely rich in RNA metabolism and protein synthesis pathways in DN samples (Fig. 3D). The BIOCARTA, KEGG, and WIKI pathways were also conducted and results are shown in Fig. 3E.

### Immune characteristics of DN

**Fig. 4:**
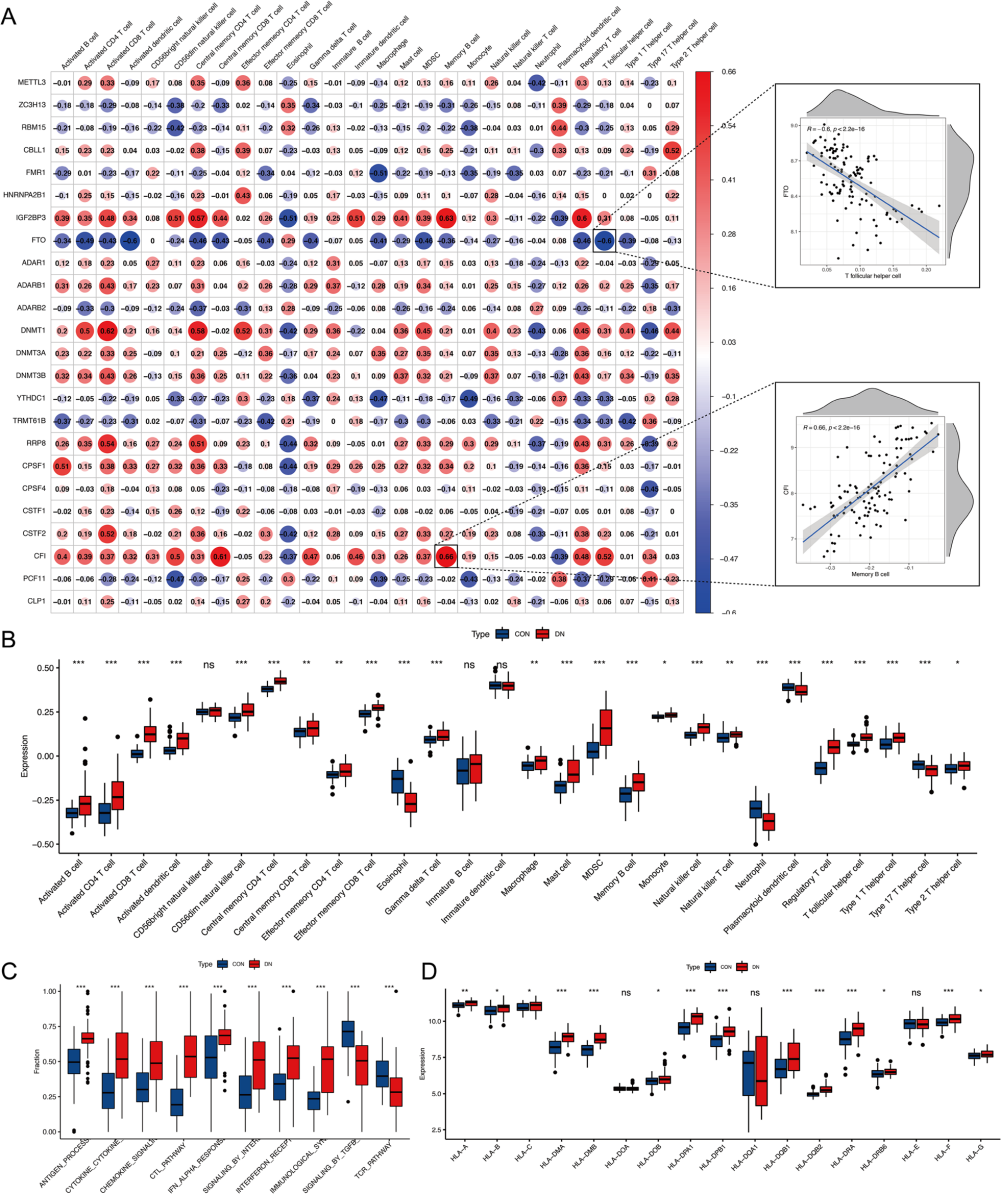
Immune characteristics in diabetic nephropathy. **A** Spearman’s rank correlation analyses between 24 RNA methylation regulators and immune-infiltrating cells at the transcriptional level, in which red represent positive correlations and blue represent negative correlations. The scatter plot shows the correlation between RNA methylation regulators and immunocyte as the significantly correlations. **B** Difference distributions of 28 immune-infiltrating cells between control and diabetic nephropathy samples. **C** Difference distributions of 10 immune reaction pathways between control and diabetic nephropathy samples. **D** Difference expression of 17 HLA alleles between control and diabetic nephropathy samples

To explore the inflammation effects on pathogenesis and progression of DN, we constructed immune characteristics analysis includes immune cell infiltrating, immune reaction pathways, HLA gene expression (Additional file 2: Tables S4–S6), and their correlations with RNA methylation regulators. We characterized the immune response with ssGSEA to visualize the relative abundances of 28 immune-infiltrating cells between DN and control samples. The results demonstrated significant differences in immune-infiltrating cells between the two groups except CD56bright NK cells, immature B cells, and immature dendritic cell. Comparing with control group, the abundance of eosinophil, neutrophil, and type 17 T helper cells were lower while the rest of immune cells were higher in DN samples (Fig. 4B). Then, ten vital immune reaction pathways were analyzed and identified that most of the immune reaction pathways of DN were markedly augmented, while TGFβ Family Members Signaling and TCR_Pathway were decreased (Fig. 4C). Subsequent analysis demonstrated that HLA gene expressions were significantly higher in DN group which suggested an active immune response and inflammation involved in DN pathogenesis (Fig. 4D).

We further investigated the association between each RNA methylation regulator and immune cells infiltration by using Spearman’s correlation analyses (Fig. 4A). Enhanced immunocyte infiltration was positively associated with elevated expression of IGF2BP3, DNMT1, and CFI, in which, IGF2BP3 was highly correlated with memory B cell, regulatory T cell, central memory CD8 T cell, CD56dim natural killer cell and immature dendritic cell with correlation coefficient values over 0.5. YTHDC1, TRMT61B, and PCF11 exhibited negative correlations with the immune infiltration level. m5C-regulator DNMT1 was mediating the activation of CD8 + T cells and central memory CD4 + T cells. CFI showed significant positive effects on memory B cell and central memory CD8 T cell with the correlation coefficient values of 0.66 and 0.61, respectively. Correlation analysis between the immune reaction pathways and RNA methylation regulators were performed (Additional file 1: Fig. S2A). We found DNMT1 was positively correlated with CTL_Pathyway whereas IGF2BP3 was negatively correlated with TGFβ Family Members Signaling. High expression of METTL3, DNMT1, and RRP8 was significantly associated with HLA gene sets such as HLA-A, HLA-B, HLA-C, HLA-DMA, HLA-DMB (Additional file 1: Fig. S2B). This indicated HLA types may predispose to related to the immune response through multiple mechanisms such as through changes in the expression or stability of RNA methylation.

### Clustering and construction a risk model for predictive

**Fig. 5:**
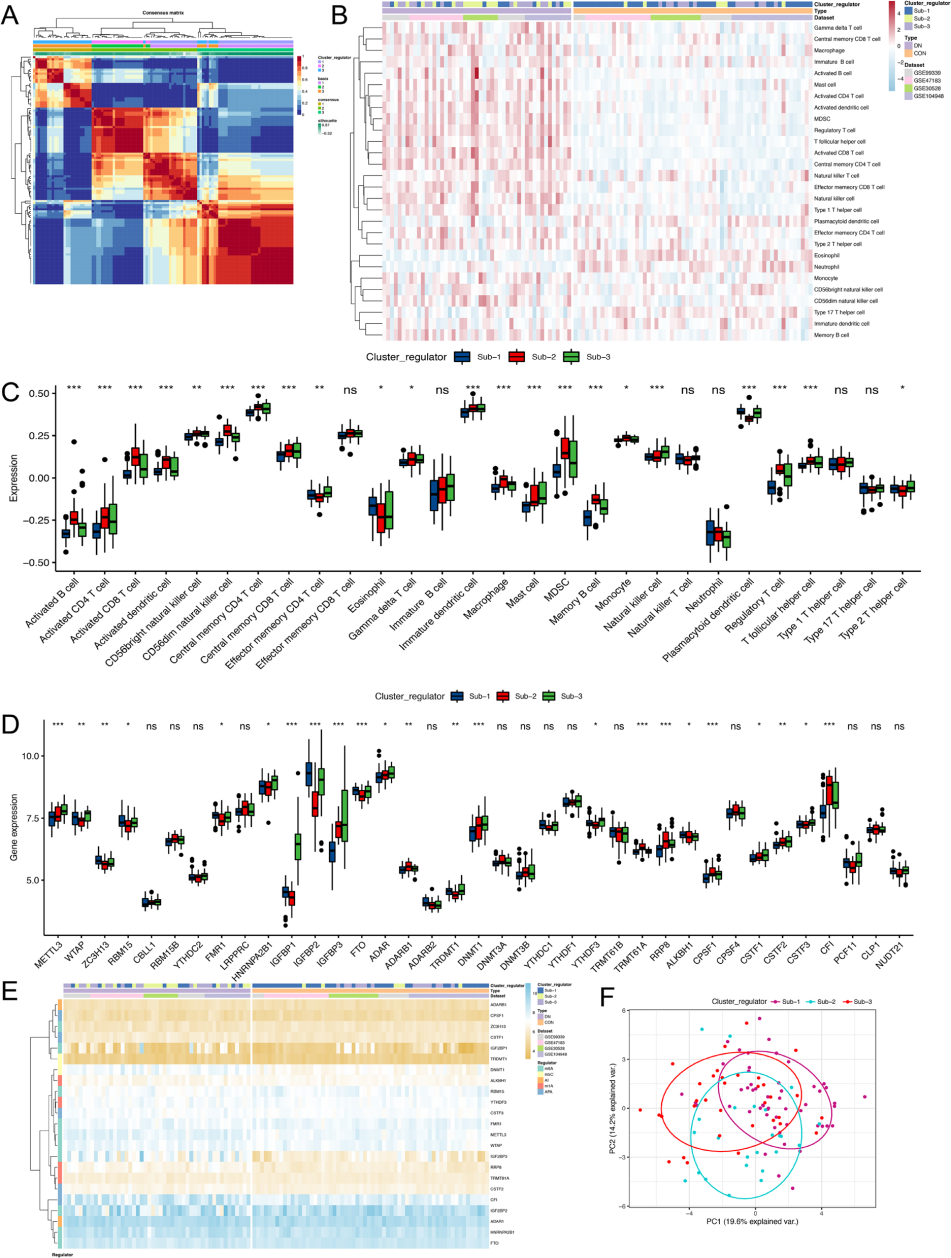
Immune characteristics in distinct RNA modification patterns. **A** Consensus matrix of NMF algorithm for *n* = 3. **B** Unsupervised clustering of 37 RNA methylation regulators in the meta-cohort. Clinical information including Type and GEO database, as well as the cluster subtype, were shown in annotations above. Red represented the high expression of regulators and blue represented the low expression. **C** The fraction of immune-infiltrating cells in three clusters using the ssGSEA algorithm. *P < 0.05; **P < 0.01; ***P < 0.001. **D** Difference expression of each RNA methylation regulators in three modification subtypes. **E** Difference expression levels of RNA methylation regulators in three modification subtypes. **F** Principal component analysis for the expression of three subtypes

To further investigate RNA modification patterns in DN, nonnegative matrix factorization (NMF) algorithm was conducted and the results are shown in Additional file 1: Fig. S3. The three subtypes based on 37 RNA methylation regulators and their relationships with immune-infiltrating cells are shown in Fig. 5A,B. The immune-infiltrating cells were significantly different among three clusters (Fig. 5C). Subtype-1 performed relatively low infiltrated immunocytes compared with Subtype-2 and 3. Subtype-2 had highest level of infiltrated activated B cells, activated CD4 T cells, CD56dim natural killer cell, macrophage, MDSC, while activated CD4 T cell, eosinophil, mast cell, and nature killer cells were enriched in Subtype-3. This new categorization was able to distinguish RNA methylation regulators based on gene expression at the transcriptional level (Fig. 5D,E), demonstrated the diversity of epigenetic modification existed in DN. Three distinct subtypes of DN were identified, including 53 samples in Subtype-1, 24 samples in Subtype-2 and 34 samples in Subtype-3 (Fig. 5F).

**Fig. 6:**
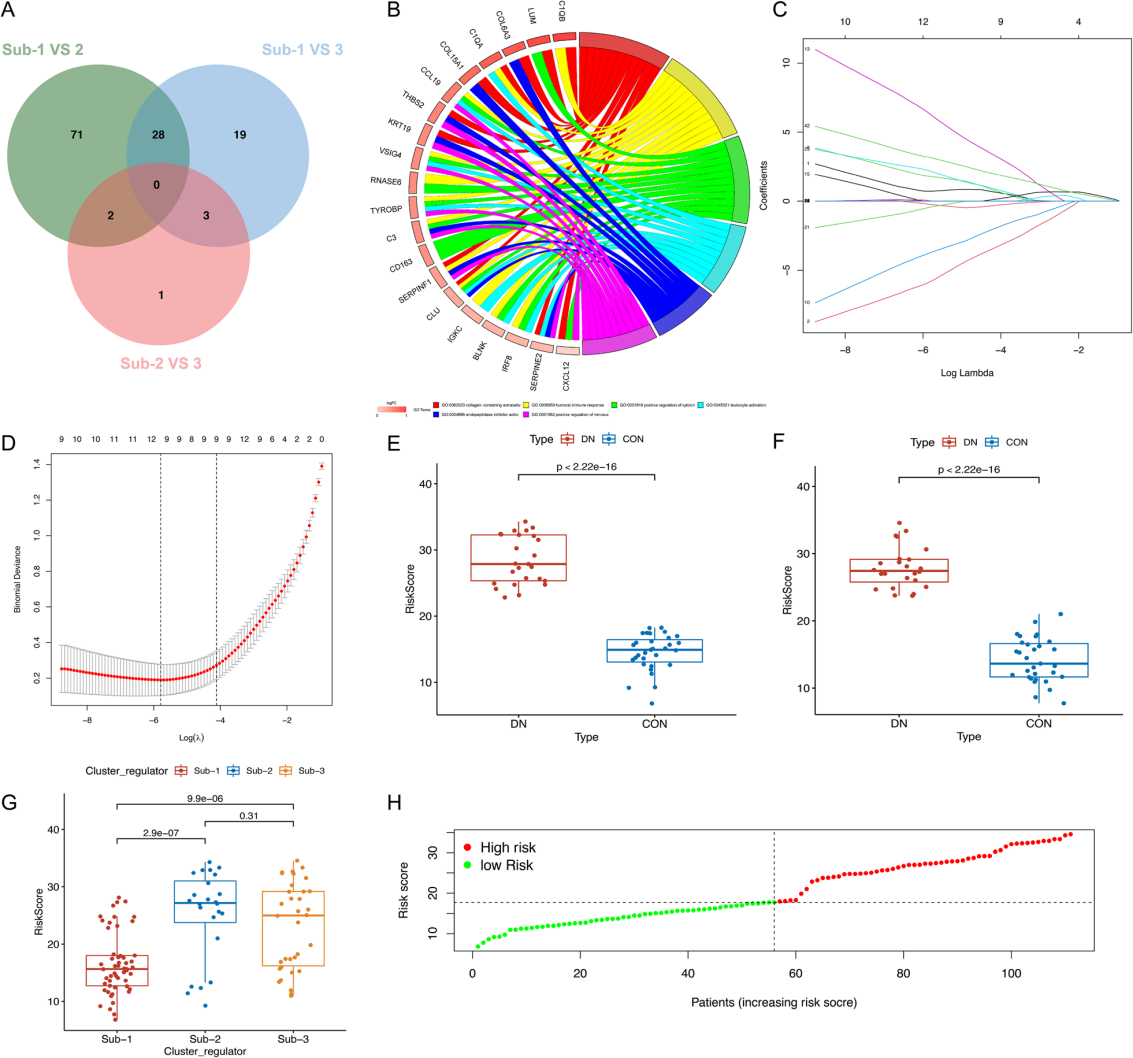
Construction of differential expression of methylation gene signatures and functional annotation. **A** 28 RNA methylation-related differentially expressed genes (DEGs) between three subtypes were shown in the Venn diagram. **B** Functional annotation for RNA methylation-related genes using GO enrichment analysis. **C** LASSO coefficient profiles of 52 RNA methylation regulatory genes to verifying the optimal Lambda. **D** Ten coefficients were selected in the LASSO regression. **E** The risk score distribution of training set in control and diabetic nephropathy samples. **F** The validation of risk score distribution of testing set in control and diabetic nephropathy samples. **G** Distribution of risk score among distinct RNA modification subtypes. **H** The risk score profiles for each patient and divided into high- and low-risk group according the median cut-off value (17.961)

Then, to explore the underlying genetic alterations within these phenotypes, a total of 28 overlapping differentially expression genes (DEGs) which represented critical identified of three RNA modification patterns were illustrated by Venn diagram (Fig. 6A, Additional file 2: Table S7). GO enrichment analysis of these signature genes revealed that biological processes related to collagen-containing extracellular, humoral immune response, positive regulation of cytokine, and leukocyte activation (Fig. 6B). These results further demonstrated that the overlapped RNA related DEGs had also related to immunity, which were consistent with our previous results. Based on 28 identified RNA methylation-related DEGs and 24 RNA modification regulators, we constructed a novel risk model to quantify the impact of RNA methylation regulatory genes on the alteration of all cases. Univariate logistic regression was used to identify the risk factors of DN and screened 43 genes for the subsequent analysis (Additional file 2: Table S8). Then, we used “caret” R package randomly classify the patients into training (*n* = 56) and testing (*n* = 55) groups at a ratio of 1:1. LASSO regression was performed and 10 RNA regulatory genes were remained according to the minimum partial likelihood deviance (Fig. 6C,D). In summary, 6 RNA methylation regulators (METTL3, RBM15, DNMT3B, TRMT61B, CPSF4, PCF11) and 4 methylation-related genes (BLNK, MS4A4A, CXCL12, COL15A1) were conducted to construct the risk model. Then, the risk score for each patient was calculated and patients with risk scores lower than the median risk score (cut-off value 17.961) were categorized into low-risk group whereas those with scores above median were placed in high-risk group (Additional file 2: Table S9). The risk scores of DN samples were significantly higher than controls in both training (Fig. 6E) and testing (Fig. 6F) datasets, indicated that high-risk score was more prompt to progress to DN condition. Compared with Subtype-1, Subtype-2 had a significantly higher risk score (Fig. 6G). However, the small sample of Subtype-2 and 3 and the shortage of constructed risk model might lead to there is no difference between the Subtype-2 and Subtype-3. The distributions of risk scores in each patient are shown in Fig. 6H.

### Evaluation of RNA methylation regulators and immune characteristics between the high- and low-risk groups

**Fig. 7:**
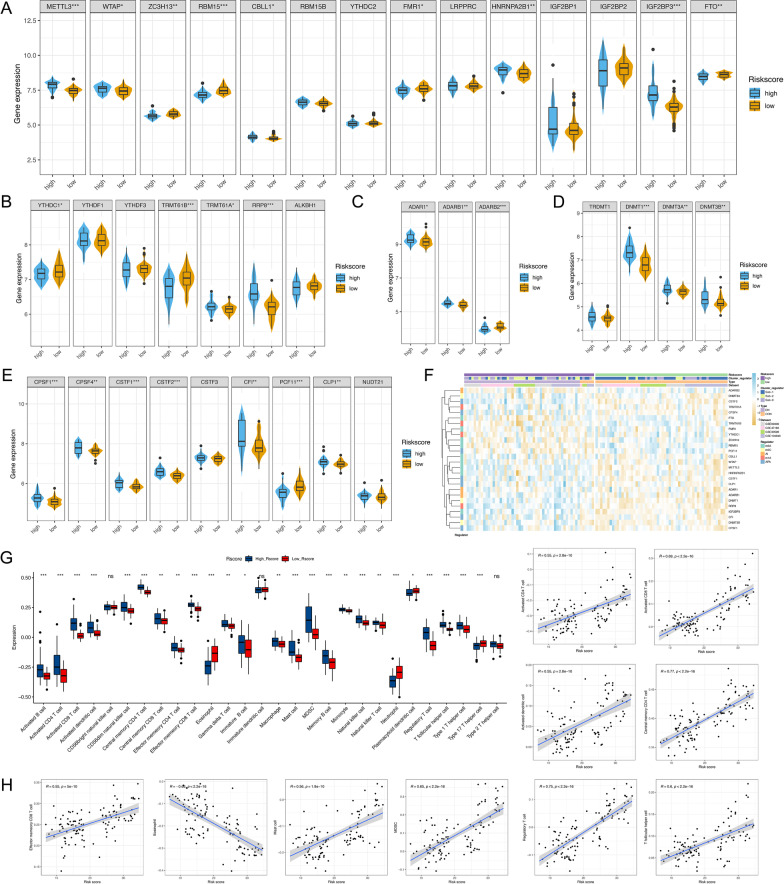
Identification and function analysis of riskScore model. **A** The violin plot demonstrated the expression of 14 m6A regulators between high-risk and low-risk groups. **B** Expression status of 7 m1A regulators. **C** Expression status of 3 A to I editing regulators. **D** Expression status of 4 m5C regulators. **E** Expression status of 9 APA regulators. **F** Difference in features and expression levels of RNA methylation regulators between high-risk and low-risk groups. **G** The fraction of immune-infiltrating cells in high-risk and low-risk groups. *P < 0.05; **P < 0.01; ***P < 0.001. **H** Ten immunocyte were significantly correlate with risk score by spearman analysis

To further demonstrate the characteristics of the risk model, we compared the expression of RNA methylation regulators between high- and low-risk group. Significant differences in RNA gene profiles were observed and this result was in accordance with the difference between DN and control group, which suggested our risk model had the robust capability to distinguish DN and control samples (Fig. 7F). As shown in Fig. 7A, METTL3, ZC3H13, CBLL1, HNRNPA2B1, IGF2BP3 were highly expressed in high-risk group, whereas FTO RBM15, FMR1 were highly expressed in low-risk group. For m5C, APA, m1A, A-I methylation type, the results are shown in Fig. 7B–E, respectively. Then, we utilized ssGSEA algorithm to assess the association between riskScore and the abundance of immune-infiltrating cells and found that the high-risk group was characterized by increasing immune infiltration (Fig. 7G). The risk score was positively correlated with higher expression of T cells such as activated CD4 T cells, activated CD8 T cells, regulatory T cells, and T follicular helper cells, whereas negatively correlated with eosinophil, as indicated in the scatter diagrams (Fig. 7H). The relationships between the risk score and B linage cells such as active B cells, memory B cells, macrophage, were weak (Additional file 1: Fig. S4A). We also assessed the relationship between the ten risk genes in the proposed model and the abundance of immune cells. Results shown that most immune cells were significantly correlated with the COL15A1, BLNK, MS4A4A, CXCL12 genes (Additional file 1: Fig. S4B). Furthermore, we sought to determine the predictive ability of the risk model in HLA gene. As expected, patients in high-risk group were significantly associated with a high expression of HLA (Additional file 1: Fig. S4C). This phenomenon of HLA-related protection against immunity could conceivably involve several mechanisms and then immune reaction pathways analysis reveal that multiple biological processes were remarkably related to the high-riskscore group patients (Additional file 1: Fig. S4D). Combining both, a potential association of HLA polymorphisms with DN-related autoimmune, implicating this HLA gene in this complication of diabetes mellitus.

### Validation of functional phenotypes in human podocyte cell lines

**Fig. 8:**
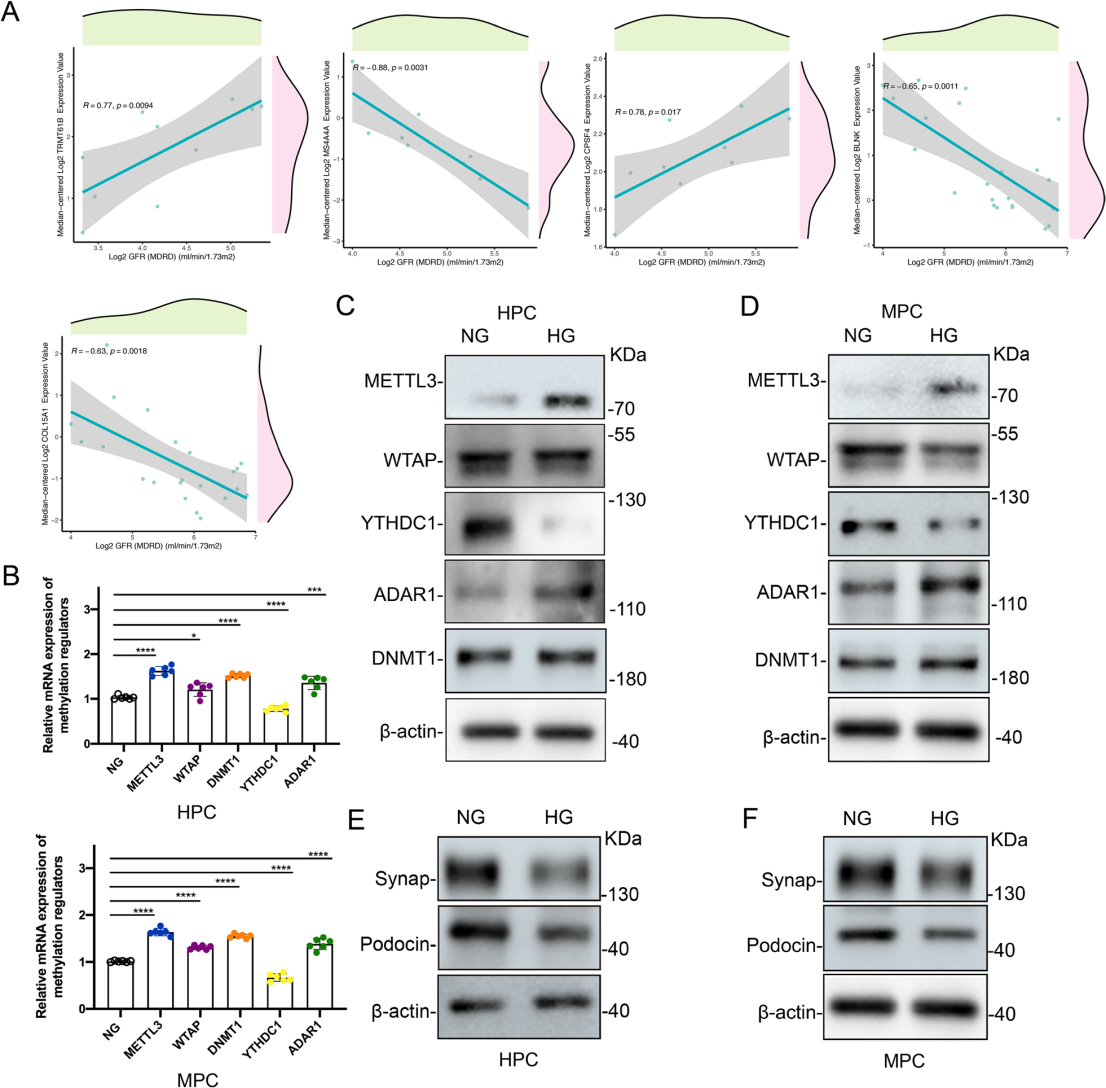
Validation of functional phenotypes in database and cell lines. (**A**) Correlations between five RNA methylation regulatory genes and eGFR. (**B**) Relative expression levels of METTL3, WTAP, YTHDC1, ADAR1, DNMT1 mRNA in immortalized human podocyte cell line (HPC) and mouse podocyte cell line (MPC) by qRT-PCR. *P < 0.05; **P < 0.01; ***P < 0.001; ****P < 0.0001 versus normal glucose (NG) group. (**C**, **D**) Immunoblot analysis of RNA methylation regulators protein in high glucose (HG) induced podocytes. (**E**, **F**) Immunoblot analysis of podocyte damage-related protein in high glucose (HG) induced podocytes.β-actin served as a loading control

**Fig. 9:**
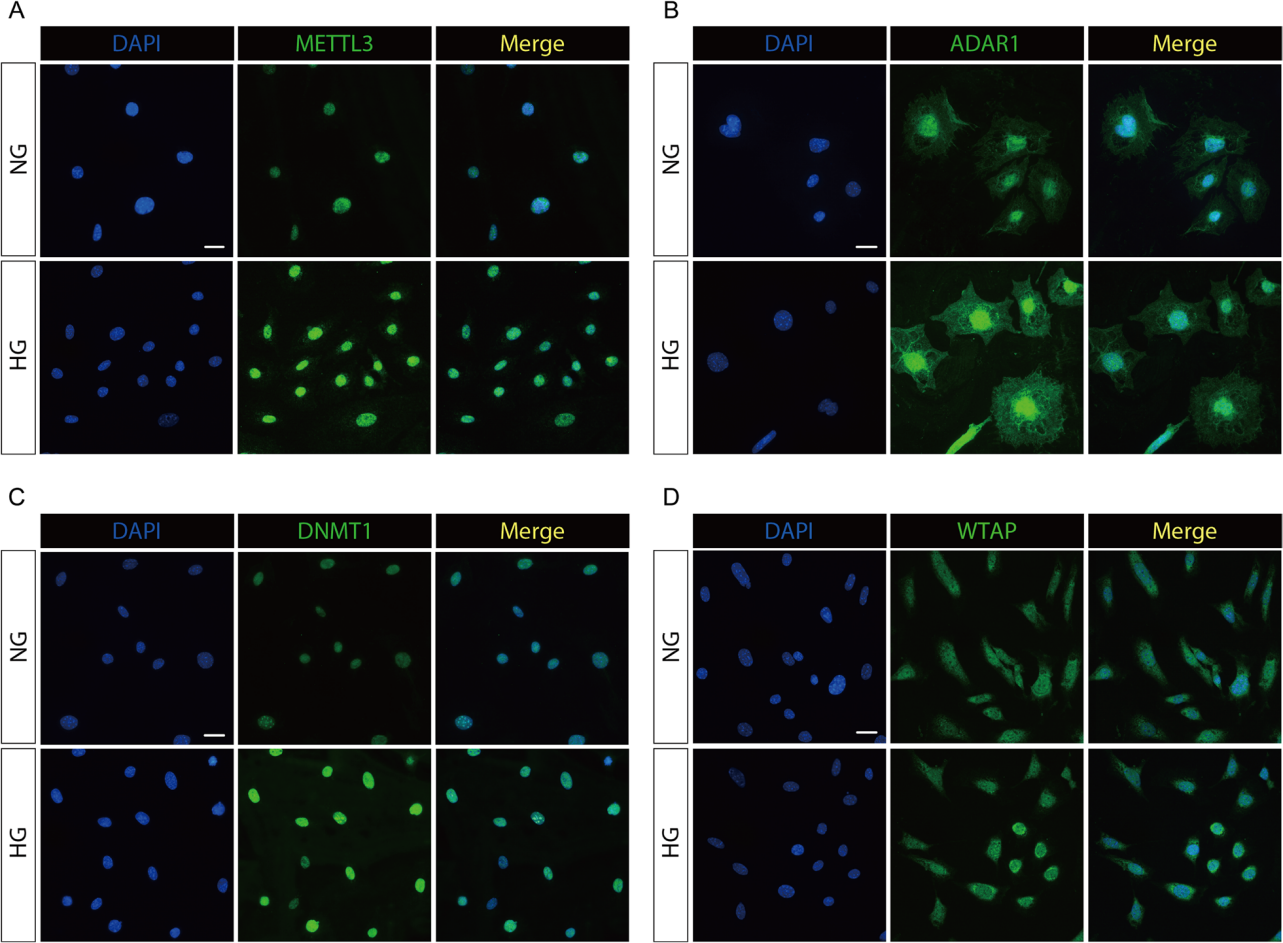
Representative immunofluorescence staining for RNA methylation regulators (green) and DAPI (blue) in different groups of human podocytes. (**A**) Immunofluorescence co-localization was used to analysis the expression of METTL3, ADAR1 (**B**), DNMT1 (**C**) and WTAP (**D**). Scale bars: 10 μm. HG, high glucose, 30 mM; NG, normal glucose, 5.5 mM

To elucidate the roles of our risk model genes, we investigated the correlation between ten genes and eGFR in the Nephroseq Database. Unfortunately, five of them were removed in further analyses since they were not currently involved in the database. Among the remaining five genes, TRMT61B and CPSF4 was positively correlated with eGFR, which means higher expression indicates better renal function in DN patients (Fig. 8A). The BLNK, MS4A4A, and COL15A1 were all negatively correlated with eGFR, indicating that these genes may aggravate kidney damage in patients with DN. Then, we verified the expression of METTL3, WTAP, YTHDC1, ADAR1, DNMT1 protein in immortalized human podocyte cell line (HPC) and mouse podocyte cell line (MPC) by qRT-PCR and Western blot. Both the HPC and MPC expressed the high levels of METTL3, ADAR1, DNMT1 proteins whereas the expression of YTHDC1 was expressed at obviously low levels as compared with the NG condition (Fig. 8C,D). The expression of podocyte damage-related protein, podocin, and synaptopodin, decreased in HG condition (Fig. 8E,F). The intracellular localization of METTL3 WTAP, YTHDC1, ADAR1, DNMT1 was visualized by immunofluorescence localization, as shown in Fig. 9.

## Discussion

The pathophysiology of diabetic nephropathy is complicated and involves interactions between genetic and epigenetic factors. Currently transcription factors have not been fully efficacious in the progression of DN, suggesting that further understanding of the epigenetic mechanisms is necessary for the improved management of this disease. With the rapid growing RNA research in the burgeoning field of RNA methylation, it becomes clear that these modifications play vital roles on modulating gene expression and controlling cell death, thereby igniting the new insights in RNA-based therapeutic strategies. As such, it is crucial to investigate the renal expression of these differentially methylated genes in diabetic individuals. In our current study, we firstly identified the expression profiles of RNA methylation modifications with special emphasis on m6A, m5C, m1A, APA, A-I editing, and their effects on immune microenvironment in DN patients. By explaining the regulation pattern of gene expression through post-transcriptional modification, our results provide novel scientific evidences for understanding the pathogenesis of DN.

To well understand the relationship between RNA modification and DN, the catalytic mediators of its “writer”, “eraser,” and “reader” proteins need to be firstly interpret. There were 37 RNA methylation regulators, in which, 24 of them were significant differently expressed in DN patients, suggesting the involvement in DN pathogenesis. Most regulators showed close interactions in PPI network revealing the cooperative regulation of RNA methylation. Among these 24 regulators, METTL3, DNMT1, ADAR1, RRP8, and CSTF2 from m6A, m5C, A to I, m1A, and APA exhibited the highest fold change values in DN patients, respectively. METTL3, acting as a “writer,” was found to be a methylation regulator inhibiting the expression of HHLA2 in renal cell carcinoma [24]. However, activated HHLA2 could relieve inflammation microenvironment by reducing interleukin-2 and interferon-γ production and T cell proliferation [25]. In our current study, METTL3 performed highly correlations with activated CD8 T cell, central memory CD4 T cell and effector memory CD4 T cell, suggesting the stimulation of T cells may contribute to development of DN via inflammation pathway. YTHDC1, known as m6A readers, is also necessary for its recognition of m1A [26]. ADAR1 is recognized in adenosine-to-inosine RNA conversion and prevent double-stranded RNA (dsRNA) damage and regulates innate immunity and the interferon-mediated response [27]. The above three chemical modification of RNA structure were all occur in adenine, but m5C is particularly added S-adenosyl-methionine (SAM) to the carbon-5 position of the cytosine base in untranslated RNA region [28]. APA is involved in the 3’end cleavage and polyadenylation and dysregulated frequently leading to changes in oncogenes and tumor invasion metastasis [29].

Next, to gain further insight into the immune characteristics of glomeruli in DN patients, we identified the expression of immune-infiltrating cells and HLA gene sets. The results shown that CD4 memory T cells, follicular helper T cells, T regulatory cells (Treg), gamma delta T cells, and neutrophils play pathogenic roles in immune defense of DN. Eller et al. found that depleting Treg cells with anti-CD25 antibody in db/db mice could exacerbate diabetic renal injury including elevated albuminuria and glomerular hyperfiltration, transplanting CD4^+^FoxP3^+^cells into mice could alleviate it, which was accordance with our results [30]. Emerging evidence indicated that inflammation played a critical role on pathogenesis of DN, such as inflammatory cytokines, tumor necrosis factor (TNF)-α, and immune mediators in serum or peripheral blood cells [31]. In our study, we identified the expression levels of activated B cells, memory B cells, monocyte and macrophage were significantly increased in DN group. Previous studies also indicated the activation of B cells, monocytes and macrophages were contributed to progression of DN via antigen presentation and abnormal antibody sedimentation [32].

The correlations between RNA regulators and immune characteristics were close, implying that RNA modification also contributed to the immune microenvironment regulation. We found that DNMT1 and FTO were highly correlated with activated CD8 + T cells and T follicular helper cells, respectively. DNMT1 potentiated T cell recruitment and deletion of DNMT1 in double-positive thymocytes leading to the activated T cells proliferation impaired [33, 34]. Up-regulating DNMT1 in diabetic immune cells could generate abnormal cytosine methylation of upstream regulators of mammalian target of rapamycin, following with aggravated inflammation in diabetic renal tissue [35]. Fat mass and obesity-associated (FTO) was responsible for the demethylation of m6A modifications and also mediates demethylation of m1A in tRNA [36]. In papillary thyroid cancer, FTO acts as a tumor suppressor via suppress glycolysis and cell growth through IGF2BP2-mediated m6A modification [37].

Although there was limited study reported the association between HLA-gene sets and RNA methylations in DN. The aberrant expressions of HLA were contributed to different inflammation consequences in various immune-related diseases. In diabetic patients, the HLA-DQB1*0501 allele showed protective effects on the progression of diabetic kidney disease in a Chinese Han population [38]. On the other hand, in a Canadian T2DM population, comparing with other types of HLA, the HLA-A2 with either HLA-DR4 or HLA-DR8 were associated with development of ESRD in younger age [39]. These findings highlight the complexity in assessing the potential role of HLA in DN. Advances in molecular techniques and the use of more samples are needed for the improved understanding of HLA alleles associations with DN.

Then, we evaluate the biological pathways of RNA modification types. DN samples were differently enriched in HALLMARKS pathway, especially glycolysis, epithelial-mesenchymal transition and oxidative phosphorylation. Zeng et al. reported that glucose dysregulation can initiate macrophage glycolysis and the release of inflammatory factors, causing renal injury in mice [40]. Reduced activity of glycolytic enzymes, oxidative phosphorylation, and increased production of reactive oxygen species is a principal link in the occurrence and progression of DN [41]. In addition, we specifically analyzed REACTOME pathways that concentrate on the exact mechanistic detail involved in RNA methylation. The epigenetic regulation of rRNA expression and diseases of programmed cell death pathways were precisely located in RNA metabolism and protein synthesis biological pathways in DN samples. In this sense, our results provided new evidence to reveal the pathological and immunotherapy management in DN glomerular injury.

We identified three distinct RNA methylation modification patterns by nonnegative matrix factorization clustering of the DN samples. Each subtype characterized by different expression of RNA methylation regulators and immune characteristics. Sub-2 and Sub-3 patterns were associated with elevated activated infiltrating immunocytes and innate immune cells such as neutrophils and macrophages. Macrophages were demonstrated to be the most prevalent infiltrating leucocytes in diabetic kidneys and associated with declining renal function in DN patients [42]. Then, we adopted an algorithm strategy and construct a risk model to quantify the contributions of RNA methylation regulatory genes to the prognosis of every case in the training set. The RNA modification pattern characterized by the immune-high phenotype exhibited a higher risk score, while the pattern characterized by the immune-low phenotype showed a lower risk score. Considering the high-risk group were mostly DN patients, the risk score could be capable of being a predictive biomarker in DN and associated with immune reaction. We identified the correlation between the risk score and immunocytes and the results suggesting that the riskScore system could applied in DN patients to determine immune characteristics and therapeutic targeting of the innate immune system.

Besides elucidated the results of riskScore model, we also explored the ten RNA methylation regulatory genes in Nephroseq Database. TRMT61B was responsible for 1-methyladenosine of mitochondrial tRNA and participated in altered gene expression which involved in mitochondrial processes in Alzheimer’s disease [43, 44]. CPSF4 protein functionally regulated the transcription of specific target RNAs and could generate a broad range of oncoproteins for its mRNA export and translation activities [45]. High CPSF4 expression was positively associated with eGFR in DN patients. BLNK as a central linker protein, regulating biological outcomes of B cell function and development [46]. We identified BLNK were negatively correlated with eGFR and this is consistent with results that DN samples present a higher number of activated B cells and memory B cells than controls, which indicated that B cell depletion might be most beneficial in individuals at high risk of DN.

To validate the results of our bioinformatic analyses, we proved that METTL3, ADAR1 and DNMT1 were all expression in DN cell lines and higher expression when compared with normal glucose condition. YTHDC1 expression was downregulated in HPC and MPC. The expression of DNMT1 and WTAP were not significantly different in HPC, whereas DNMT1 expression was increased and WTAP were downregulated in MPC. These all localizes with speckles in interphase nuclei and associated with nuclear pre-mRNA splicing components [47].

Nevertheless, our study had some limitations. Although our study primarily reveals the expression of serval RNA regulators in vitro, its functions and metabolic network are required further intensive laboratory work. The series of new identified RNA regulators were not obtained from the databases and high throughput sequencing methylation profiling are needed for further verification. Besides, it was difficult to obtain datasets that simultaneously included clinical data, the small sample size of the GEO row data and the shortcoming of the constructed riskScore model might lack of prognostic value. A larger number of clinical data and modification of the risk model are still required to evaluate the clinical correlations.

## Conclusions

In conclusion, our study is the first comprehensive approach by integrating epigenetic and genetic signals to identify novel disease-driving pathways and therapeutic targets. We reveal that the aberrant expression of RNA methylation regulators and immune infiltration regulation play critical roles in the pathogenesis of DN, which could promote predictive-based DN management. Greater understanding of the relationship between RNA methylation regulators and kidney function has the potential not only to further the understanding of immune renal disease at a fundamental level but also to lead to the development and application of more effective, specific and less toxic therapies for DN treatment.

## Supplementary Information

**Additional file 1. Figure S1.** Batch normalization of four datasets to remove batch effects. **Figure S2.** Immune microenvironment characteristics in diabetic nephropathy. **Figure S3.** Unsupervised clustering of 52 RNA methylation regulatory genes in the meta-GEO cohort. **Figure S4.** Immune characteristics between high- and low-risk groups.

**Additional file 2. Table S1.** The primer sequences for target mRNA. **Table S2.** The baseline information of included individuals in four GEO cohorts. **Table S3.** Correlation analysis of the 24 RNA methylation regulators. **Table S4.** Immune cell infiltration characteristics of diabetic nephropathy samples in eight GEO cohorts. **Table S5.** Enrichment score of immune reaction pathways in four GEO cohorts. **Table S6.** Enrichment score of HLA gene set in four GEO cohorts. **Table S7.** Differentially expressed genes between Subtype_1, Subtype_2, and Subtype_3. **Table S8.** The univariate logistic regression analysis to identified the risk genes of diabetic nephropathy. **Table S9.** Samples clustering in four GEO cohorts.

## Data Availability

The data used to support the findings of this study are included within the article.

## Abbreviations

A–I: Adenosine-to-inosine
APA: Alternative polyadenylation
DM: Diabetes mellitus
DN: Diabetic nephropathy
DEGs: Differentially expression genes
ESRD: End-stage renal disease
eGFR: Estimated glomerular filtration rate
GEO: Gene expression omnibus
GSVA: Gene set variation analysis
HLA: Human leukocyte antigen
HPC: Human podocyte cell line
LASSO: Least absolute shrinkage and selection operator
m5C: 5-Methylcytosine
MPC: Mouse podocyte cell line
m1A: N1-methyladenosine
m6A: N6-methyladenosine
NMF: Nonnegative matrix factorization
RAAS: Renin–angiotensin–aldosterone system
ssGSEA: Single-sample gene set enrichment analysis
