# Supplementary material for: Integrated analysis of RNA methylation regulators crosstalk and immune infiltration for predictive and personalized therapy of diabetic nephropathy
Source: Hum Genomics. 2023 Feb 10;17:6. doi: 10.1186/s40246-023-00457-9 (PMC9912588; doi:10.1186/s40246-023-00457-9)
Supplement: Supplementary file 1 — Additional file 1. Figure S1. Batch normalization of four datasets to remove batch effects. Figure S2. Immune microenvironment characteristics in diabetic nephropathy. Figure S3. Unsupervised clustering of 52 RNA methylation regulatory genes in the meta-GEO cohort. Figure S4. Immune characteristics between high- and low-risk groups. [file 40246_2023_457_MOESM1_ESM.docx]

**Figure S1.** Batch normalization of four datasets to remove batch effects.





**Figure S2. Immune microenvironment characteristics in diabetic nephropathy.** (A) Spearman’s rank correlation analyses between 24 RNA methylation regulators and immune reaction pathways, in which red represent positive correlations and blue represent negative correlations. The most positive and negative correlated pair is shown in two scatterplots. (B) The correlation between 24 RNA methylation regulators and HLA gene expression. The most positive and negative correlated pair is shown in two scatterplots.





**Figure S3. Unsupervised clustering of 52 RNA methylation regulatory genes in the meta-GEO cohort.** (A) The distribution between cophenetic, dispersion, residuals, and silhouette coefficients with respect to the number of clusters. (B) Heatmap of NMF clustering for RNA methylation regulatory genes in 5 meta-GEO with cluster numbers from 2 to 9.





**Figure S4. Immune characteristics between high and low-risk groups.** (A) Relationships between the risk score and immune cells. (B) Relationships between the ten riskScore genes in the proposed model and the abundance of immune cells. (C) The expression differences of HLA alleles in high and low-risk groups. (D) The fraction of immune response pathways in different risk score groups. *P < 0.05; **P < 0.01; ***P < 0.001.
